# Vibrational spectral analysis, XRD-structure, computation, exo⇔endo isomerization and non-linear optical crystal of 5-((5-chloro-1*H*-indol-2-yl)methylene)-1,3-diethyl-2-thioxodihy-dropyrimidine-4,6 (1*H*,5*H*)-dione

**DOI:** 10.1186/s13065-019-0524-8

**Published:** 2019-02-02

**Authors:** Mezna Saleh Altowyan, Assem Barakat, Abdullah Mohammed Al-Majid, Hazem A. Ghabbour, Abdelkader Zarrouk, Ismail Warad

**Affiliations:** 1 Department of Chemistry, College of Science, Princess Nourah Bint Abdulrahman University, Riyadh, Saudi Arabia; 2 Department of Chemistry, College of Science, King Saud University, P.O. Box 2455, Riyadh, 11451 Saudi Arabia; 3 Department of Chemistry, Faculty of Science, Alexandria University, P.O. Box 426, Ibrahimia, Alexandria, 21321 Egypt; 4 Department of Pharmaceutical Chemistry, College of Pharmacy, King Saud University, P.O. Box 2457, Riyadh, 11451 Saudi Arabia; 5 Department of Medicinal Chemistry, Faculty of Pharmacy, Mansoura University, Mansoura, 35516 Egypt; 6 Laboratory of Materials, Nanotechnology and Environment, Faculty of Science, Mohammed V University, 4Av. IbnBattuta, B.P. 1014 Rabat, Morocco; 7 Department of Chemistry, Science College, An-Najah National University, P.O. Box 7, Nablus, Palestine

**Keywords:** Condensation, Thiobarbituric acid, Exo–endo isomer, XRD

## Abstract

This work deals with the synthesis and characterization of the novel 5-((5-chloro-1*H*-indol-2-yl)methylene)-1,3-diethyl-2-thioxodihydro-pyrimidine-4,6(1*H*,5*H*)-dione π-bridge (D–A–D) donor–acceptor–donor compound. Its exo-isomer structure has been proven by XRD-single-crystal analysis for the first time. The IR, UV–Vis., MS, CHN-, ^1^H and ^13^C NMR analysis were also carried out. The DFT-optimized structural-parameters were matched with the XRD-crystallographic data. The experimental-XRD-interactions in the lattice were compared to the computed Hirshfeld analysis (HSA), MEP map and Mulliken charge population. The DFT/6-311G(d) calculations like IR/B3LYP, TD-SCF, HOMO–LUMO, GRD and GIAO-NMR have been compared to their corresponding experimental parameters. Non-linear optical (NLO) crystal theoretical-analysis was carried out then compared to urea reference. The compound thermal activity was evaluated in an open-atmosphere by TG/DTG analysis.
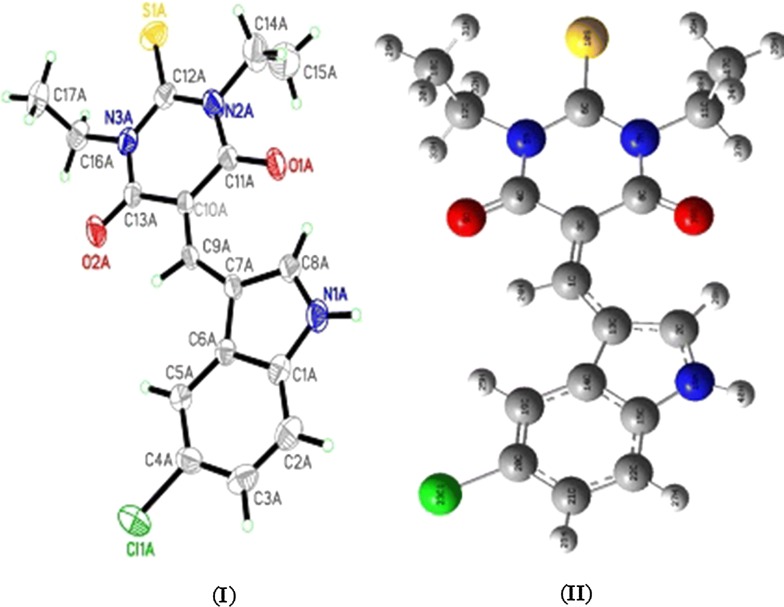

## Background

Barbituric acid and thiobarbituric acid and their derivatives as hypnotic-compounds containing the active methylene are considered being as a good starting material to prepare specific class of heterocyclic molecules via Knoevenagel mild condensation condition [1–4]. Combination of thiobarbituric acid and different aldehydes via dehydration reactions is a useful synthetic technique to design novel mono-/or poly-substituted thiobarbiturate derivatives [2–8]. Such compounds recently become highly attractive to pharmaceutical chemists, since it is biological very activity, it used as: anticancer, anti-inflammatory, antioxidant, antibacterial, anti-convulsing, antifungal, antihypnotics and antiangiogenic agents [5–14]. Moreover, these compounds were broadly used as enzyme inhibitors [15], for example, it was good to inhabit tyrosinase enzyme which contributed to the neurodegeneration associated with Parkinson’s disease [15–17]. For such reasons, there is an urgent need to develop novel and active tyrosinase inhibitors; which is considered as a promising breakthrough enzyme-inhibitors compounds.

Many polar-organic crystalline molecules with non-centrosymmetric crystal structures reflected a very high second-order non-linear optical (NLO) properties [18, 19]. Several organic compounds which were prepared through condensation reactions may own NLO-properties; such properties can be enhanced via introducing of π-bridge in between two different functional groups donor–acceptor–donor (D–A–D) in the desired organic compounds [19].

In this study, 5-((5-chloro-1*H*-indol-2-yl)methylene)-1,3-diethyl-2-thioxodihydro-pyrimidine-4,6 (1*H*,5*H*)-dione compound has been prepared through one pot condensation reaction in a good yield, the structure of exo-isomer was confirmed by XRD-single crystal and spectrally characterized. Several experimental spectral measurements were compared with their corresponding theoretical parameters. Initially, exo–endo isomerization reaction was DFT-computed and its T.S was detected under QTS2 level of calculation.

## Results and discussion

### Synthesis

**Scheme 1:**
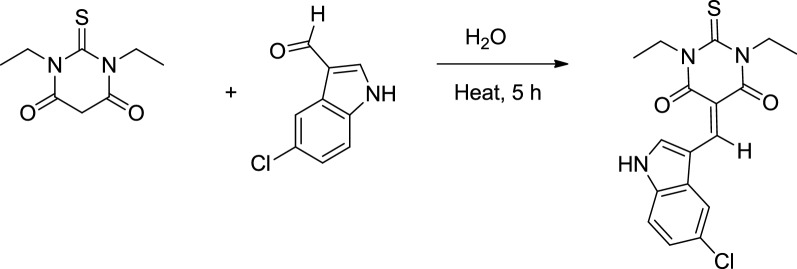
Synthesis of desired thiobarbiturate product

Linking thiobarbituric acid with suitable aldehydes in order to prepare heterocyclic thiobarbiturate derivatives for structural analysis and pharmaceutical applications become a very broad area of research [1–3]. The 5-((5-chloro-1*H*-indol-2-yl)methylene)-1,3-diethyl-2-thioxodihydro-pyrimidine-4,6(1*H*,5*H*)-dione derivative was prepared through one pot Knoevenagel dehydration reaction, as in Scheme 1. Condensation of 1,3-diethyl-2-thioxodihydro-pyrimidine-4,6(1*H*,5*H*)-dione with 5-chloro-1*H*-indole-2-carbaldehyde under reflux conditions revealed the formation of the desired thiobarbiturate in a very good yield. The synthetic methodology here reflected high yield without side products, moreover, short refluxed time was required compared to recent synthetic methodologies [1–5]. Knoevenagel mild condensation condition consider to be fast and easy method of synthesis, therefore, it can be performed in simple lab, moreover, the desired product is attractive to pharmaceutical chemists, several medical applications like: anticancer, antioxidant, anti-inflammatory, antibacterial, antifungal, anti-convulsing and antiangiogenic agents can be evaluated in future work then compared to published applications [5–12]. The structure of exo-isomer was clearly identified by X-ray crystallography together with several physical analyses like: TOF–MS, CHN-EA, UV–visible, IR, ^1^H and ^13^C-NMR spectroscopy.

### TOF–MS analysis

TOF–MS of the desired compound reflected very good agreement with the C_17_H_16_ClN_3_O_2_S expected molecular formula; the (MH+) molecular ion peak was detected experimentally to be *m*/*z *= 361.3, since the theoretical *m*/*z* ion peak found to be 360.2, this seen is consistent with recent result [1, 21].

### X-ray crystallographic and optimized structures

**Fig. 1:**
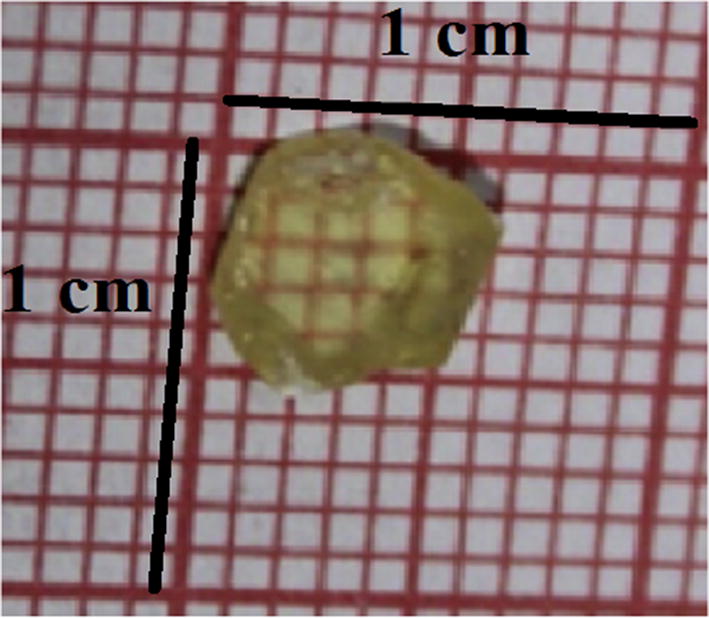
Photograph of compound crystal

Crystals were grown by slowly evaporation of ethanol from compound ethanolic solution. The XRD data was collected on BRUKER APPEX-II CCD diffractometer, using graphite monochromatic Mo Kα radiation, λ = 0.71073 Å, at T = 293(2) K. Details of crystallographic measurement are given in Table 1. Direct methods were utilized to solve the structure using SHELXS-97 program [20]. The light-yellow compound crystal is shown in Fig. 1.

**Table 1:** Summary of crystallographic data for the target compound

Parameters	
Empirical formula	C_17_H_16_ClN_3_O_2_S
Formula weight	361.84
Crystal system, space group	Triclinic, *P*-1
*Unit cell dimensions*	
*a, b, c* Å	9.1136 (3), 12.7475 (5), 15.6198 (6)
*α, γ, β* °	67.0300 (10), 81.2960 (10), 79.0530 (10)
Volume Å^3^	1634.36 (11) Å^3^
Z	4
Density (calculated) (Mg m^−3^)	1.471
Absorption coefficient (mm^−1^)	0.38
Crystal size (mm)	0.42 × 0.16 × 0.15
θ range for data collection	2.3–25.3
Reflections collected	37,028
Independent reflections	7138
Final [I > 2σ(I)]	0.064
R indices (all data) CCDC	0.0635 1,532,937

**Fig. 2:**
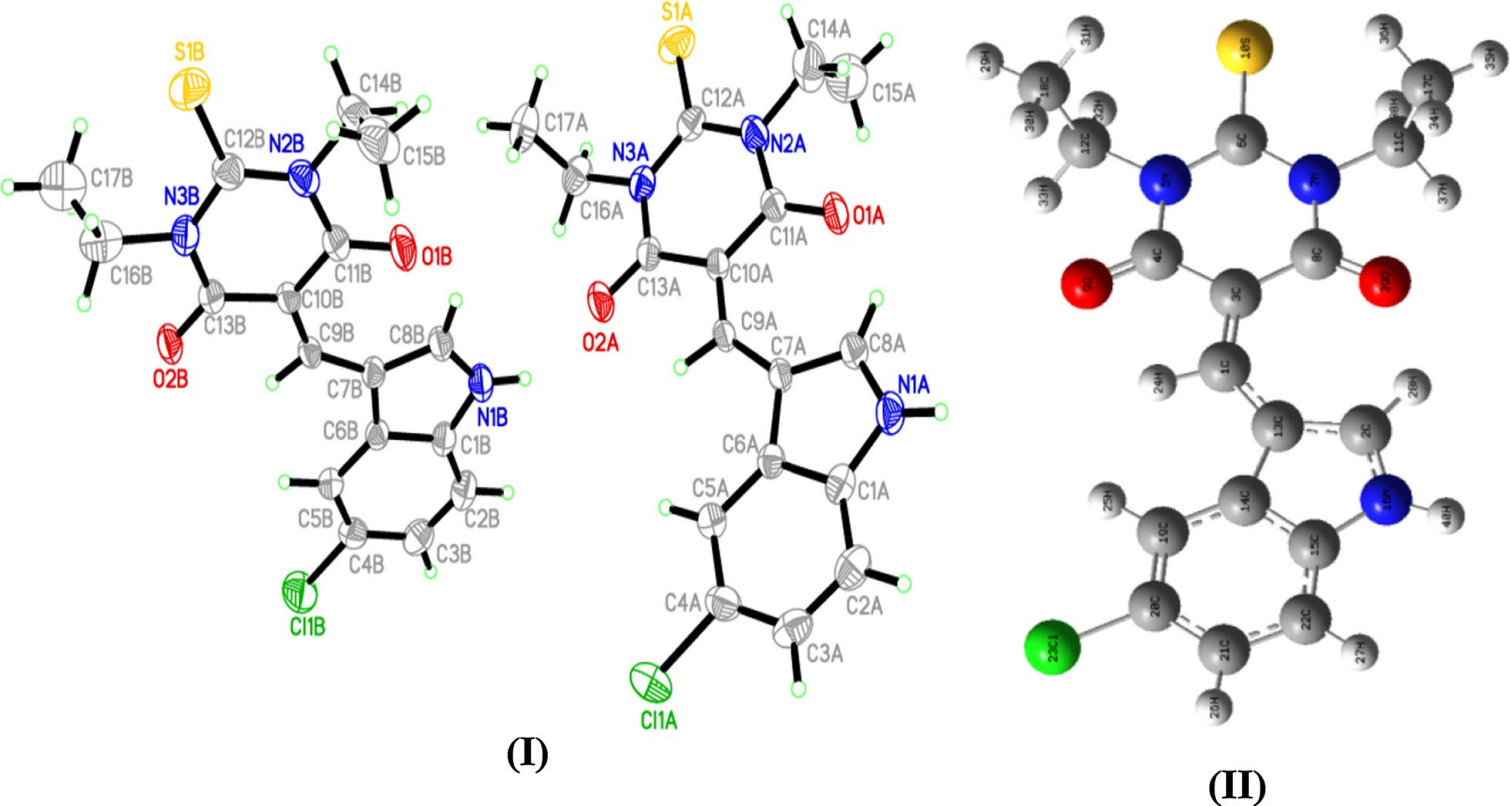
Structure of exo-isomer: **I** ORTEP and **II** B3LYP/6-311G(d) optimized structure

The structure of thiobarbiturate molecule was solved by single-crystal X-ray diffraction and computed by B3LYP/6-311G(d), as in Fig. 2. X-Ray diffraction suitable crystals were grown by recrystallization from ethanol solvent. The compound crystallizes in Triclinic with space group *P*-*1*, *Z *= 4 and cell parameters *a *= 9.1136 (3) Å, *b *= 12.7475 (5) Å, *c *= 15.6198 (6) Å, *α *= 67.0300 (10)°, *β *= 81.2960 (10)° and *γ *= 79.0530 (10)°.

In solid state, no solvent molecules were detected in the crystal lattice; the desired thiobarbiturate is composed of thioxodihydro-pyrimidine-4,6(1*H*,5*H*)-dione ring bonded to 5-chloro-1*H*-indole ring via C=C bond, the two ethyl groups which were bonded to the thioxodihydro-pyrimidine-4,6(1H,5*H*)-dione via the N atoms are in *trans* positions to each other. The two rings are in one plane which flattens the molecule, XRD structure confirmed such seen since all the atoms in the molecule (except terminal ethyl groups) are with sp^2^ hybridizations. The structure was solved as dimer, the two molecules connected together via N–H…O strong H-bond in perpendicular planes. The two molecules in the dimer are structurally semi-identical and both solved as exo-isomer stereo-structure (Fig. 2a).

The B3LYP exo-optimized parameters and XRD-structural (bond lengths and angles) are listed in Table 2.

**Table 2:** Selected experimental XRD bond lengths and angles compared to the DFT-B3LYP calculated result

Bond no.	Bond (Å)		Exp. XRD	DFT/B3LYP	Angle no.	Angles (^o^)			Exp. XRD	DFT/B3LYP
1	Cl1	C4	1.741	1.7608	1	C1	N1	C8	110.4	110.6
2	S1	C12	1.646	1.676	2	C11	N2	C12	125.1	125.02
3	O1	C11	1.227	1.2244	3	C11	N2	C14	115.6	115.02
4	O2	C13	1.217	1.2193	4	C12	N2	C14	119.2	119.94
5	N1	C1	1.381	1.3902	5	C12	N3	C13	125.1	124.76
6	N1	C8	1.333	1.3534	6	C13	N3	C16	116.3	114.96
7	N2	C11	1.391	1.4086	7	Cl1	C4	C3	117.9	118.42
8	N2	C12	1.382	1.385	8	Cl1	C4	C5	118.8	119
9	N2	C14	1.523	1.4848	9	C7	C9	C10	136.6	136.4
10	N3	C12	1.388	1.3882	10	O1	C11	N2	118.9	118.99
11	N3	C13	1.394	1.4101	11	O1	C11	C10	123.9	124.08
12	N3	C16	1.485	1.4844	12	S1	C12	N3	121.8	121.56
					13	O2	C13	N3	119.9	119.74
					14	O2	C13	C10	123.6	123.41

**Fig. 3:**
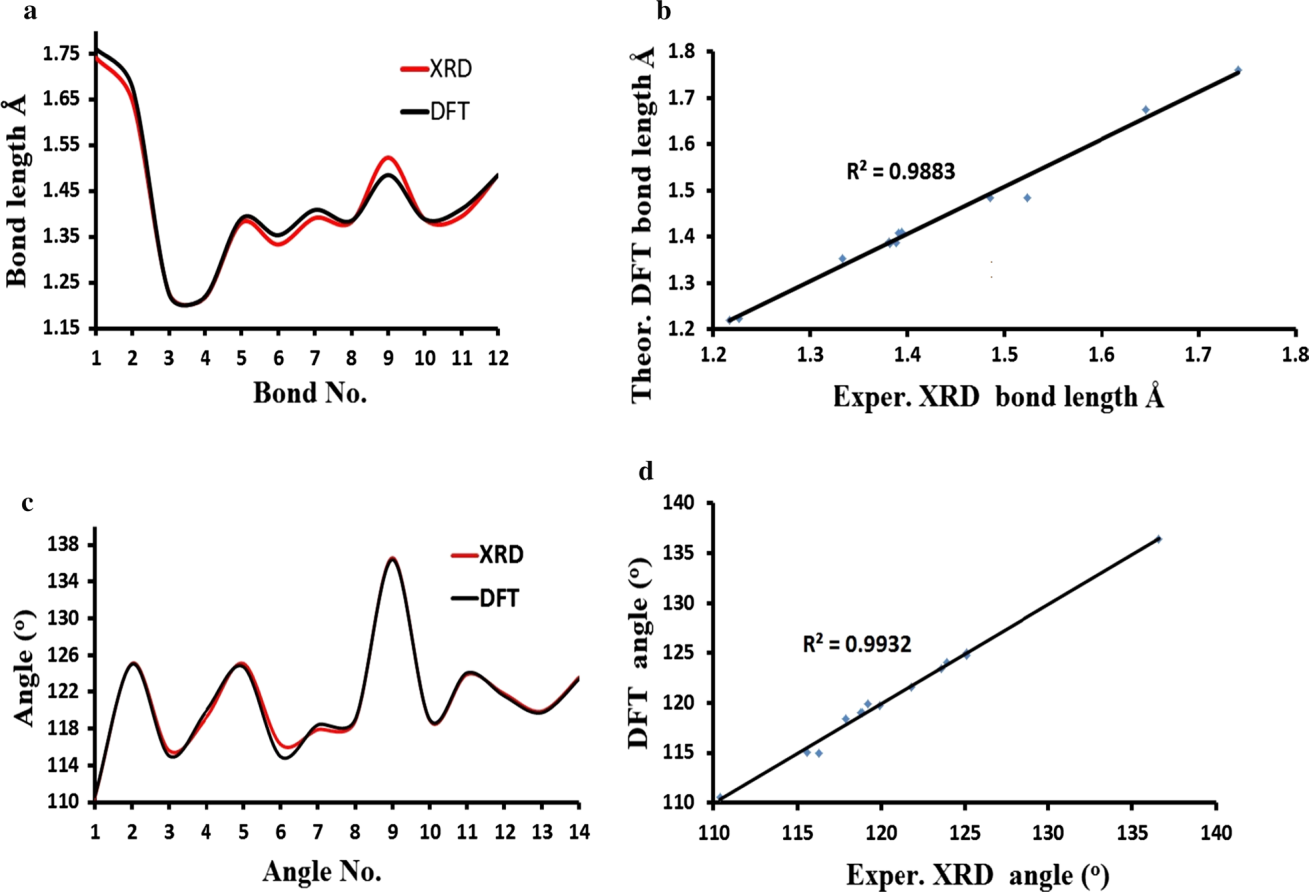
**a** Bond lengths histogram and **b** graphical correlation of exp. against DFT, **c** exp. vs. DFT angles values histogram together with its graphical correlation (**d**)

A very good matching between calculated and theoretical structural parameters results were collected, as seen in Fig. 3. The bond length vs. bonds type and the angle value vs. angles type in both experimental and theoretical are very close in their values, as seen in Fig. 3a, c. Excellent graphical correlations between the exp.-XRD and DFT/B3LYP calculated bond distances and angles were found to be 0.9883 and 0.9932, respectively (Fig. 3b, d).

### Endo/exo DFT-isomerization via sp^2^–sp^2^ single flip rotation

**Scheme 2:**
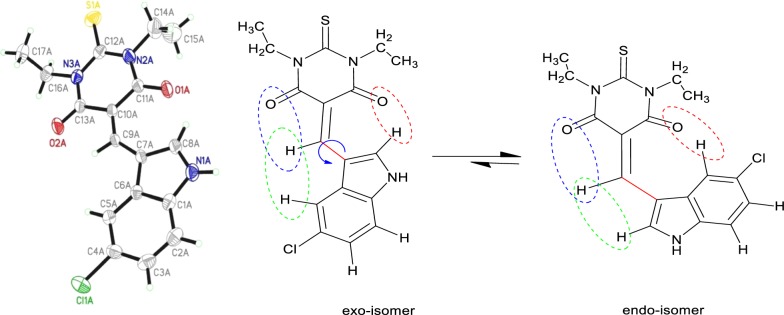
*Exo*–*endo* isomerization

Since the thiobarbituric acid is a high symmetrical organic compound, the Knoevenagel condensation reaction with aldehydes thiobarbitone products expected to have no *E/Z* isomers [21]. Based on the XRD-structure and its energy profile, the exo-isomer is considered to be structurally-favored isomer (exo-isomer steric-less compared to the endo-isomer), as explained in Scheme 2.

**Fig. 4:**
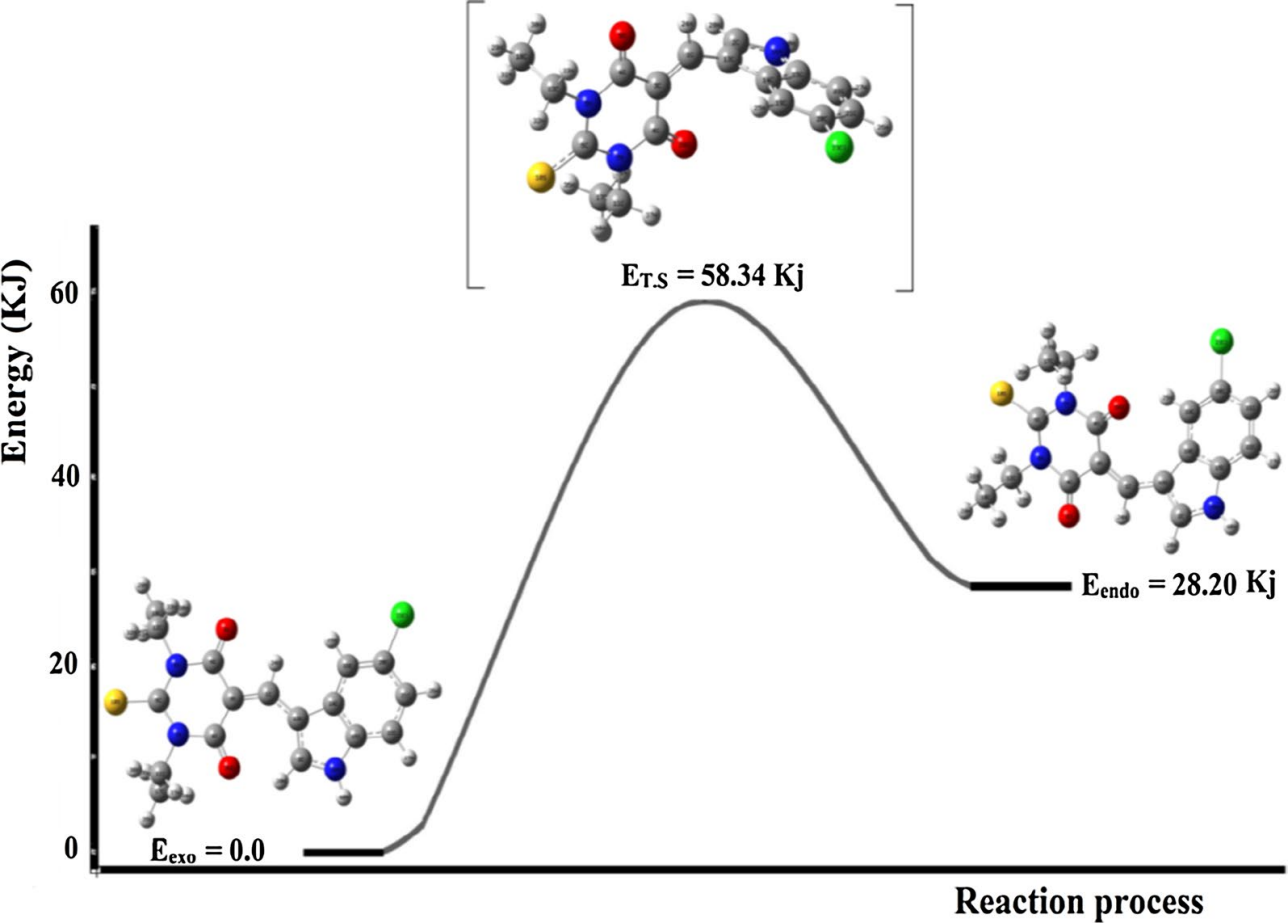
Structures and energy profiles of endo⇔ [T.S_QTS2_]⇔endo isomerization reaction

The stereo-chemical difference between the exo and endo isomers is controlled by simple flip vertical rotation around highlighted C9_sp2_–C7_sp2_ bond; this rotation caused a dramatic change in the C11–C10–C9–C7 dihedral angle from 0° (exo) to 180° (endo). Using this fact, and by neglecting all the expected intermolecular-forces in the both isomers (gaseous state), they were optimized under DFT-B3LYP/6-311++G(d) level of theory, the less global-minimum energies profile of exo-isomer (− 1830.84518316 a.u.) supported to be the more stable isomer (zero reference energy, E_exo_ = 0.0 kJ) with 1.45 dihedral angle, while for endo-isomer is with − 1830.85592406 a.u., E_endo_ = 28.2 kJ and 177.82 dihedral angle. The energy of the transition state and its structure were solved using QTS2 method of calculation. T.S energy profile found to be high the exo and endo energy − 1830.83370311 a.u. E_T.S_ = 58.34 kJ, the structure was detected as in between endo/exo isomers structure with 90° dihedral angle C11–C10–C9–C7, that is expected since two rings are in semi-perpendicular to each other. The energies profiles reflected two important seen, first the exo-isomer (less steric) is favored over endo-one (more steric) consisted with the XRD-experimental solved structure, second the rotational isomerization exo⇔endo reaction via 180° sp^2^–sp^2^ single bond flip is possible since ΔE is very small, as seen in Fig. 4.

### Combined crystal interaction, Hirshfeld, MEP and Mulliken charge analysis

**Fig. 5:**
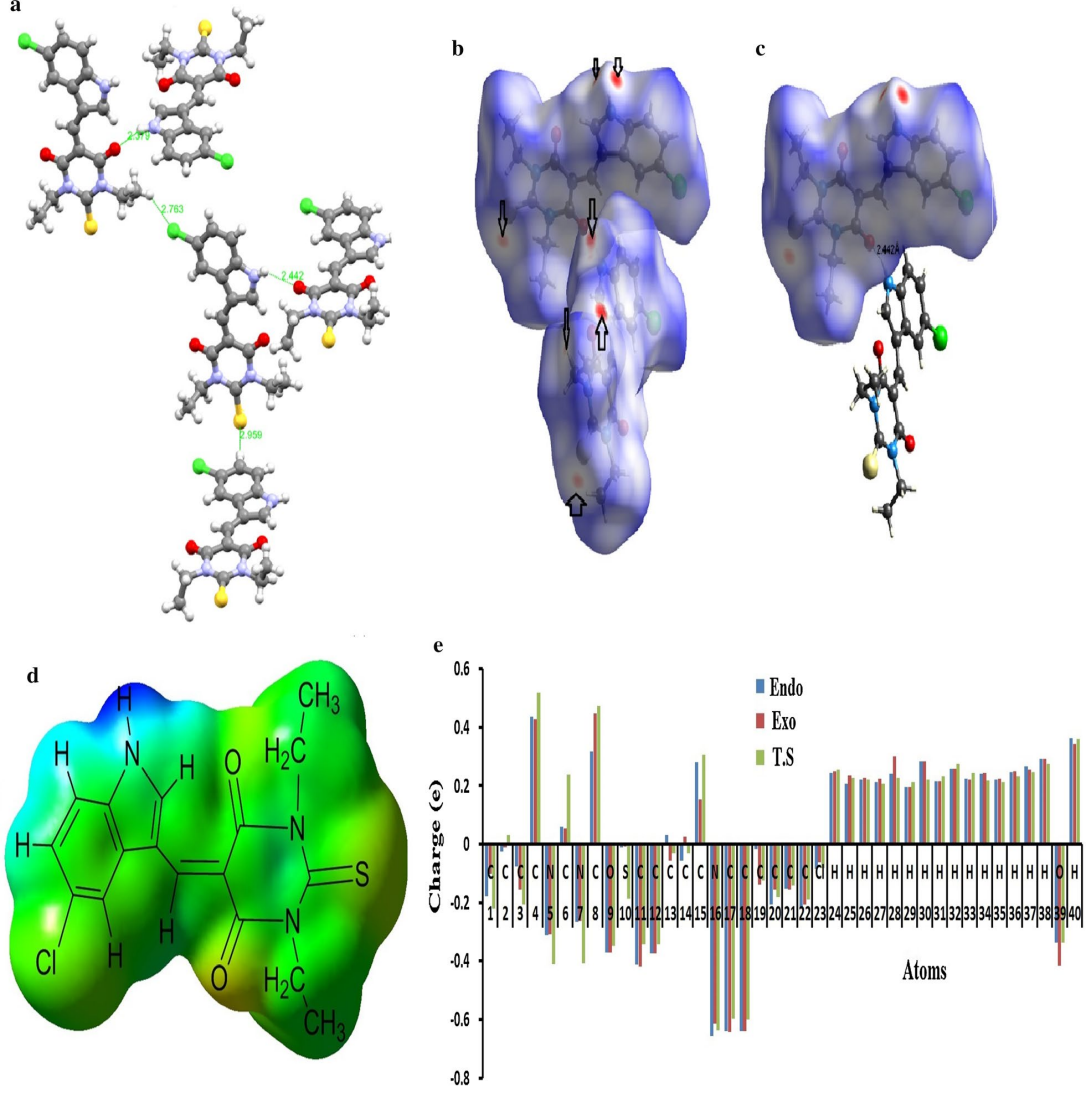
**a** XRD**-**molecular packing, **b** HSA *d*_*norm*_, **c** HSA H-bond, **d** MEP map and **e** Mulliken atomic charges

The crystal structure of the molecule was solved as dimer form (Fig. 5a); the two molecules which compose the dimer are structural semi-identical, they are nearly flat and linked together via N–H…O H-bond. The short N–H…O hydrogen bonds (2.442 Å) connected the two the molecules reflecting its stability as perpendicular bi-molecule dimer, various intermolecular short interactions such as: C–H…Cl with 2.763 Å, C–H…S with 2.959 Å and π…π with 3.348 Å were detected which stabilized crystal lattice in 3D-network morphology. The nature of these interactions was computed by HSA. The HSA result of the compound is illustrated in Fig. 5b. Since the compound contains number of heteroatoms such as S, N, O and polar H atoms it is expected to have several red-spots on the HSA computed surface [22–24]. Sufficient numbers of red-spots were detected on the molecule surface reveals the presence of H-bonds and other short contacts as seen in the *d*_*norm*_ map (Fig. 5c). The main hydrogen bonds with biggest red-point was cited N–H…O intermolecular confirming the connected of two molecules via short interaction in semi-perpendicular plane, the other H-bonds like C–H…Cl and C–H…S were detected as smaller red-points which consisted with their longer distances. Furthermore, the compound was subjected to MEP map analysis, blue-regions on the MEP surface indicated the electrophilic parts [21], for example, proton of the H–N functional group consider to be the strongest electrophile since deep blue color was observed (Fig. 5d). On the other hand, the protons of the phenyl ring are less electrophilie since light blue color was detected. The red or orange colored around S, O and Cl atoms indicated the electronic richness positions (nucleophilic functional groups). For such reason, N–H….O hydrogen bond is highly expected to be formed as main H-bond (red or orange bind blue). Such computational output is consistent with XRD-experimental collected and HSA theoretical results.

Exo, endo isomers and their T.S were subjected to DFTB3LYP/6-311G(d) Mulliken charge population analysis as summarized in Fig. 5e and Table 3. The analysis supported the existence of nucleophilic electrons-donor and electrophilic electron-acceptor functional groups in the isomers and the T.S [22]. In general all the hydrogen atoms revealed electrophilic sites in between (~ + 0.16  − 0.26e), proton of amine in exo-isomer was the highest electrophile one with ~ + 0.359e. This seen supported its acidity as well as polarity to form strong H-bonds. The carbonyl oxygen atoms in the exo-isomer showed higher nucleophilicity ~ − 0.418e explaining their roles in formation of several H-bonds in the crystal lattice of the compound. This result is consistent with the XRD packing, MPE and HSA studies.

**Table 3:** Mulliken atomic charges

No.	Atom	Endo DFT/B3LYP	Exo DFT/B3LYP	T.S DFT/B3LYP
1	C	− 0.17859	− 0.10099	− 0.22246
2	C	− 0.02482	− 0.01229	0.031003
3	C	− 0.07729	− 0.15735	− 0.20771
4	C	0.436762	0.42713	0.517251
5	N	− 0.31288	− 0.3091	− 0.41184
6	C	0.058913	0.054641	0.237828
7	N	− 0.26559	− 0.26323	− 0.40699
8	C	0.316331	0.446133	0.472488
9	O	− 0.37216	− 0.36993	− 0.34754
10	S	− 0.01272	− 0.00746	− 0.18628
11	C	− 0.41348	− 0.42001	− 0.34218
12	C	− 0.3729	− 0.37304	− 0.34273
13	C	0.030792	− 0.05752	− 0.03205
14	C	− 0.05642	0.024457	− 0.03075
15	C	0.281322	0.151964	0.305435
16	N	− 0.6557	− 0.61356	− 0.63808
17	C	− 0.63973	− 0.64329	− 0.59864
18	C	− 0.64048	− 0.64061	− 0.59967
19	C	− 0.01743	− 0.14008	− 0.12501
20	C	− 0.20775	− 0.15685	− 0.18067
21	C	− 0.1534	− 0.15567	− 0.14172
22	C	− 0.21557	− 0.20627	− 0.18907
23	Cl	− 0.08623	− 0.06326	− 0.08365
24	H	0.243713	0.249906	0.255279
25	H	0.205475	0.233417	0.226901
26	H	0.221395	0.227541	0.221877
27	H	0.212526	0.222791	0.205815
28	H	0.239113	0.299744	0.225444
29	H	0.194453	0.194734	0.213335
30	H	0.282572	0.283644	0.220557
31	H	0.214117	0.21542	0.231436
32	H	0.256058	0.25629	0.274051
33	H	0.223337	0.220546	0.244476
34	H	0.239424	0.243095	0.217952
35	H	0.219462	0.222429	0.212749
36	H	0.245473	0.248095	0.231268
37	H	0.264718	0.254106	0.245588
38	H	0.290244	0.290332	0.274633
39	O	− 0.33598	− 0.41765	− 0.33772
40	H	0.362908	0.341722	0.359372

### DFT and experimental ^1^H NMR

**Fig. 6:**
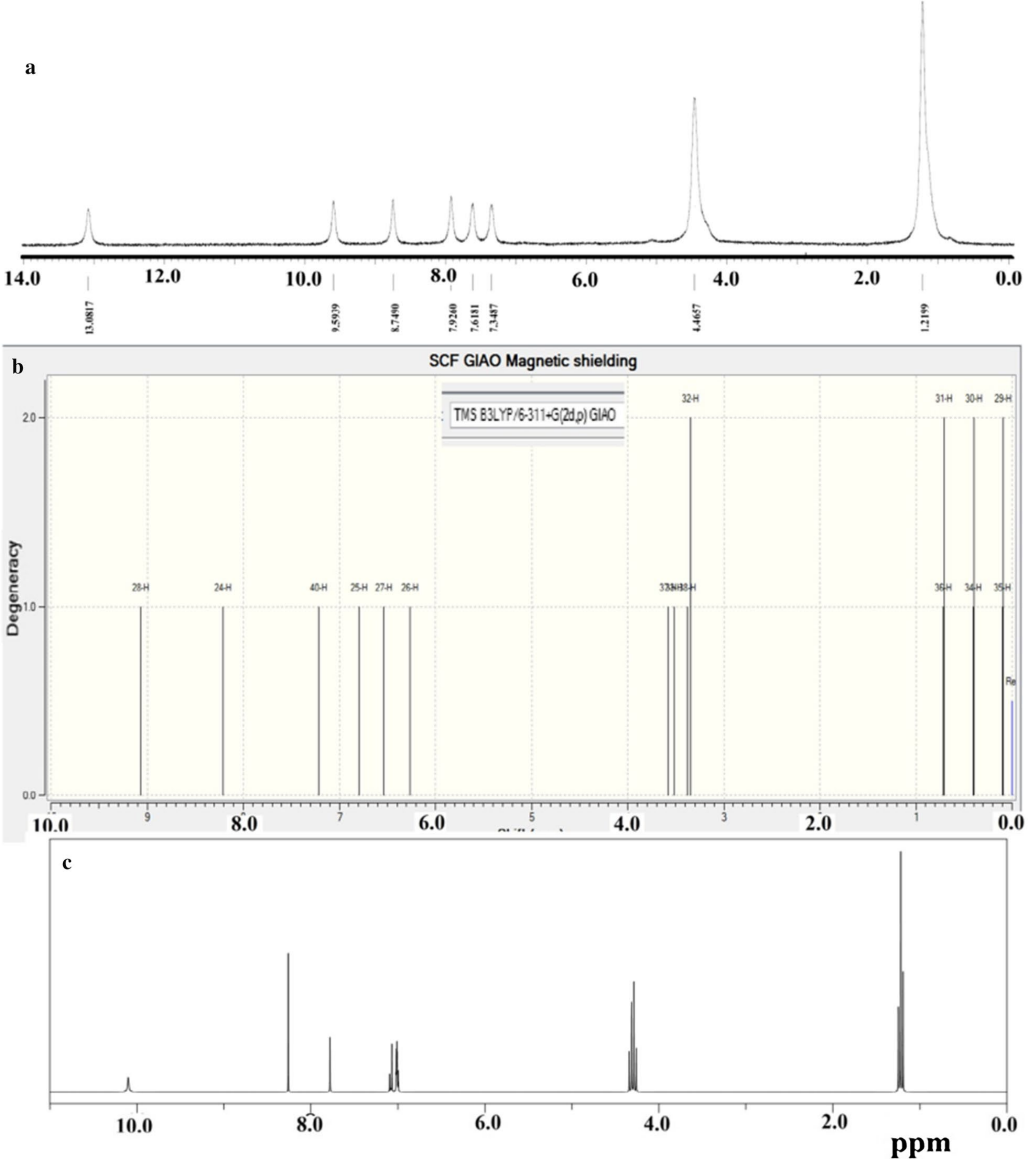
^1^H NMR spectra in DMSO-*d*_6_ **a** experimentally, and theoretically **b** GIAO, and **c** ACD-LAB programs

The theoretical and experimental ^1^H-NMR spectra of the prepared thiobarbiturate are illustrated in Fig. 6. In aliphatic region two broad peaks corresponding to CH_3_ at 1.3 ppm and CH_2_ at 4.5 ppm, four peaks corresponding to the C–H aromatic protons in between 7.0–9.0 ppm were recorded, the aldehyde-proton =CH is detected at 9.6 ppm, while the acidic amine-proton (NH) is recorded at 13.1 ppm as in Fig. 6a.

The experimental and theoretical (GIAO and ACD-LAB) in DMSO-*d*_6_ were matched as in Fig. 6b and in c. The experimental and calculated protons chemical shifts revealed an excellent correlation, the correlation coefficient determined by GIAO and ACD-LAB against experimental found to be 0.950 and 0.972, respectively.

**Fig. 7:**
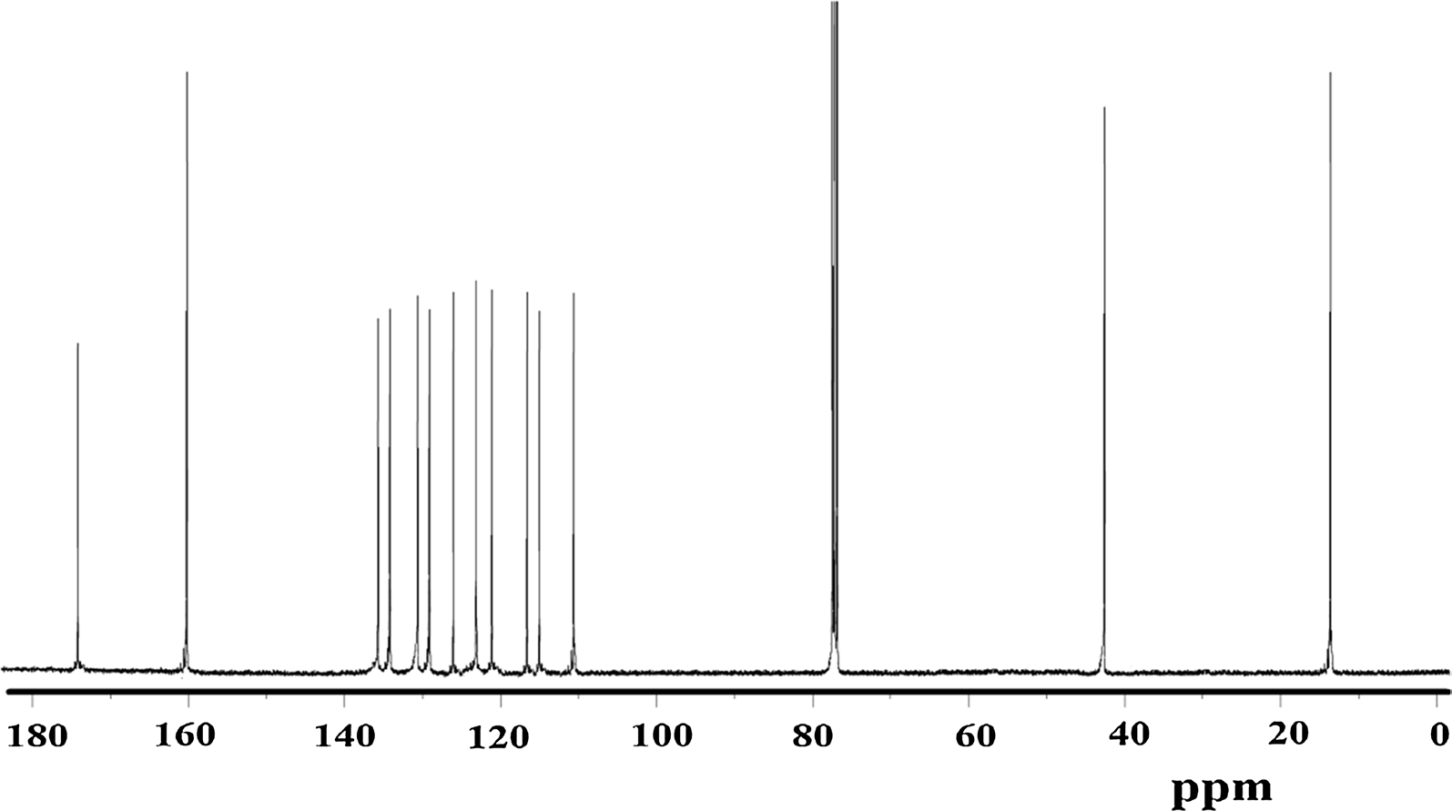
Exp. ^13^C-NMR of the compound in DMSO-*d*_6_

^13^C-NMR showed 4C’s with two-signals in the aliphatic region belongs to the CH_3_–CH_2_ were detected at 13–44 ppm, 10 C with 10 signals in the aromatic region at 110–147 ppm, 2C’s of C=O are detected at 160.8 ppm and 1C’s of C=S is detected at 173.8 ppm, as seen in Fig. 7. The ^13^C-NMR chemical shifts are compatible with the expected structure of the desired compound.

### FT-IR (DFT and experimental)

**Fig. 8:**
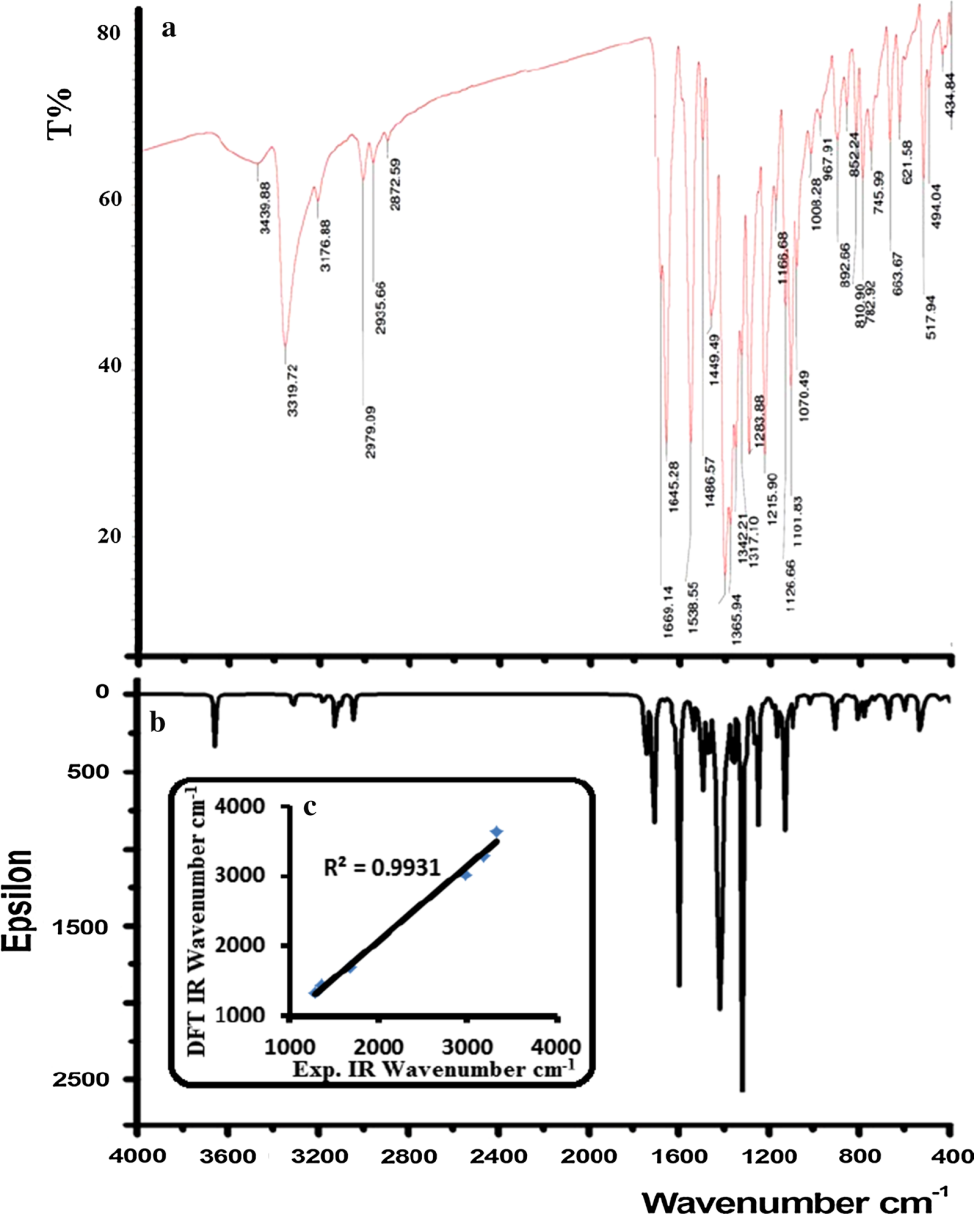
**a** Exp., **b** DFT/B3LYP/6-311G(d)-IR and **c** Exp./DFT correlation coefficient

The DFT-theoretical and experimental-IR spectra of the thiobarbiturate revealed a number of functional groups conforming its structural formula, as seen in Fig. 8. The characteristic vibrational frequencies revealed several polar functional groups like, N–H, C=O, C=S, N–C and C–Cl and nonpolar like, C–H_ph_ and C–H_alphi_, C–C and C=C. The main functional groups theoretically and experimentally chemical shifts were explained as: N–H (exp. = 3319 cm^−1^, DFT = 3635 cm^−1^), C–H_Ar_ (exp. = 3176 cm^−1^, DFT = 3290 cm^−1^), C–H_aliph_ (exp. = 2979 cm^−1^, DFT = 3020 cm^−1^), C=O (exp. = 1669 cm^−1^, DFT = 1705 cm^−1^), C=S (exp. = 1365 cm^−1^, DFT = 1440 cm^−1^) and C=C (exp. = 1286 cm^−1^, DFT = 1325 cm^−1^), the other functional group vibrations were sited to their positions [1–3, 27]. B3LYP theoretical IR frequencies are higher than experimental one, which is expected since the experimental spectra was performed in solid state while the theoretical are in gaseous state. The DFT/Exp. IR correlation coefficient found to be 0.9931 which reflected an excellent agreement between experimental and theoretical IR-analysis as seen in Fig. 8c.

### Electronic, HOMO/LUMO energy and TD-SCF transfer

**Fig. 9:**
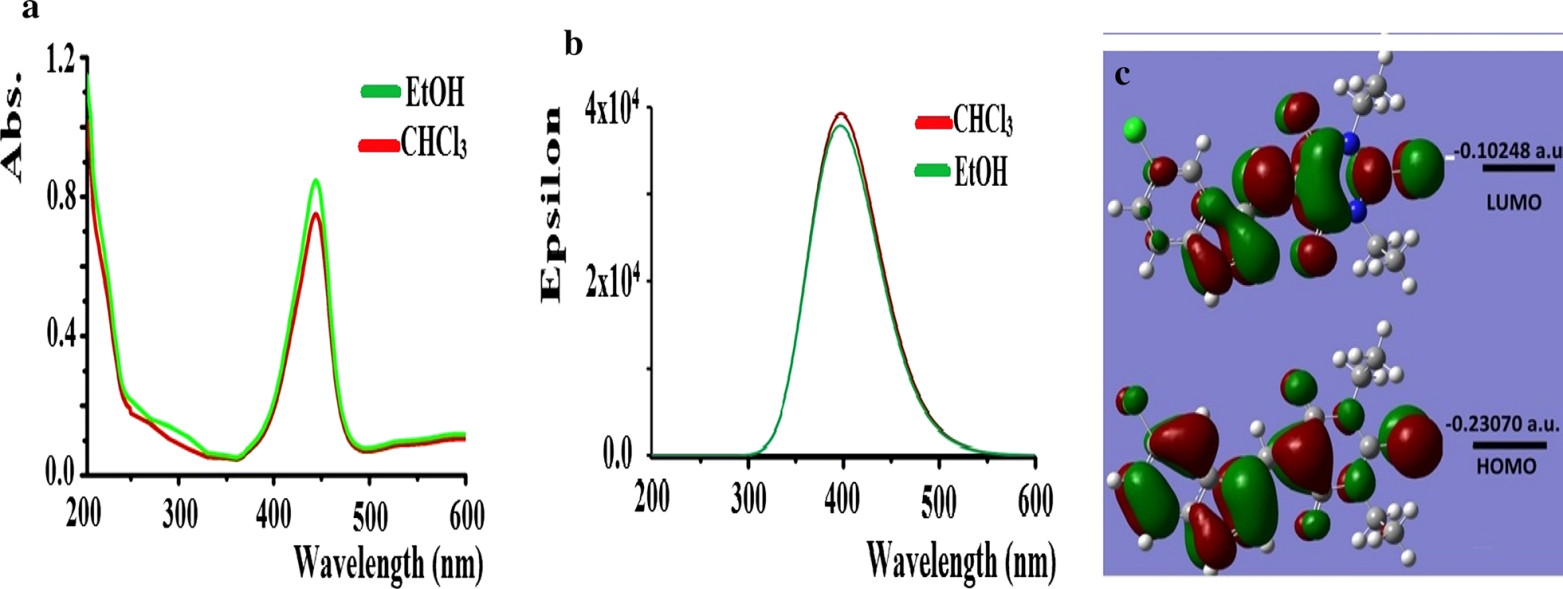
**a** Experimental UV, **b** TD-SCF/DFTB3LYP/6-311G(d) electronic spectra and **c** HOMO/LUMO shape-energy diagram in ethanol

Experimental UV and theoretical TD-SCF/DFTB3LYP/6-311G(d) spectral analysis of the desired compound were performed in ethanol and chloroform solvents, HOMO and LUMO energy levels for exo-isomer are computed in ethanol as in Fig. 9.

Experimentally, the UV behavior reflected π → π* electronic transition one sharp peak at λ_max_ = 430 nm in both ethanol and chloroform solvents (Fig. 9a). The TD-SCF/DFTB3LYP/6-311G(d) calculations exhibited one abroad band at λ_max_ = 397 nm in both solvents (Fig. 9b). No significant difference in the electronic behaviors (Uv and TD) were detected by changing solvents which reflecting a high degree of harmony between exp. and DFT analysis. The experimental wavelength showed a very good agreement with TD-B3LYP/6-311G(d) an experimentally bathochromic shift with Δλ_max_ = 33 nm were detected. To understand the electron transfer in FMO of the molecule HOMO and LUMO was computed in ethanol [25–27], ΔE_LUMO–HOMO_ = 0.128 a.u. (3.49 eV). Due to TD-B3LYP/6-311G(d) the main electron transfer at λ_max_ = 397 nm can be attributed to HOMO → LUMO (97%), while λ at 362 nm to HOMO-2 → LUMO (96%) and λ at 445 nm to HOMO-1 → LUMO + 1 (95%).

### Global reactivity descriptors (GRD)

GRD quantum parameters can be easily estimated from the energy gap levels using Koopman’s notation as follows: 
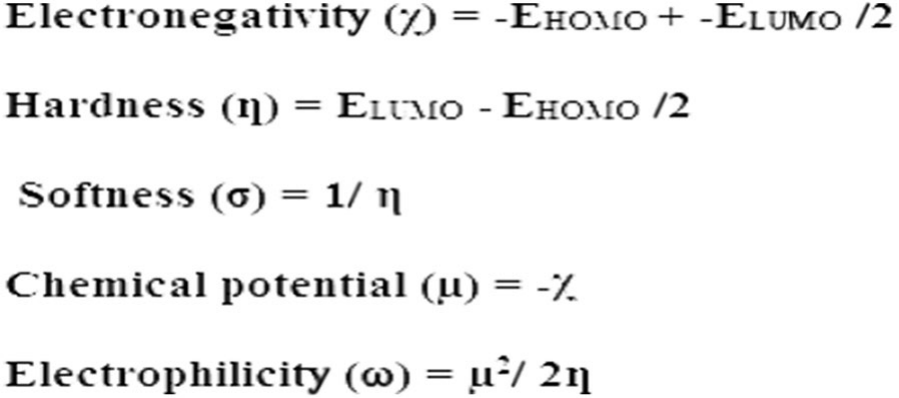


GRD parameters used by the frontier electron density to explain reactions in conjugated system and predicting the most reactive position in molecule. The conjugated-molecules are detected by a small E_HOMO/LUMO_, which facilitated the understanding of the structural activities of molecules [22–24].

Using the GRD equations above, the electronegativity (*χ*), electrophilicity (*ω*), chemical potential (µ), hardness (*ƞ*) and softness (σ) for exo-isomer were calculated, as shown in Table 4.

**Table 4:** DFT/B3LYP/6-311G(d)/GRD quantum parameters of exo-isomer in ethanol

Molecular properties	B3LYP/6-311G(d) value
E (a.u.)	− 1830.8559
E_HOMO_ (eV)	− 6.2777
E_LUMO_ (eV)	− 2.7886
ΔE (eV)	3.4890
*X* (eV)	1.74455
*ƞ* (eV)	1.74455
σ (eV)	0.5732
µ (eV)	− 1.74455
ω (eV)	0.8723
D (Debye)	6.5887

The advantage of such quantum parameters have been demonstrated to understand the molecular activities of such compound to be used as metal-coordination ligand or search for other biological applications.

### Nonlinear optical (NLO)

The quantum computation of nonlinear optical (NLO) properties of a compound has interesting role for new materials design in optical processing and modern material technology [28]. It is importance in providing optical modulation, frequency shifting, fiber, switching, optical materials laser and optical memory [22–28]. The NLO quantum calculations like polarizabilities and hyperpolarizabilities became easy and available via the DFT calculation method. Up to our knowledge, no theoretical DFT-computations were reported addressing NLO for thiobarbiturate molecules; therefore, this excited our concern to start such study. To setup the relationship between NLO and structure of the desired molecule the anisotropy polarizability (Δα) the dipole moment (μ), the mean polarizability (α) and the hyperpolarization (*β*) are calculated using B3LYP/6-31G. Urea has been used as NLO-reference proto-typical; therefore, NLO parameters of the urea were computed in the gaseous phase together with target molecule under the same level of theory, as seen in Table 5. The polarizability, anisotropy polarizability and the hyperpolarizability of title compound were calculated 16.66, 20.234 and 13.872 times better than the urea reference-molecule. It is worth noted that the 5-((5-chloro-1*H*-indol-2-yl)methylene)-1,3-diethyl-2-thioxodihydro-pyrimidine-4,6(1*H*,5*H*)-dione compound has higher NLO properties compared to urea reference, implying of such compounds to be as new NLO promising materials.

**Table 5:** The mean polarizability (α), total static-dipole moment (μ), the anisotropy polarizability (Δα) and the mean hyperpolarizability (*β*) for the studied compounds

Property	Desired compound	Urea
μx	1.3200	1.3103
μy	− 2.0651	− 1.2746
μz	0.0660	− 0.8289
μ, D	5.9861	4.1582
αxx	425.9926	31.6439
αyy	12.0326	− 11.8938
αzz	1242.0494	46.7088
αxy	− 5.9335	1.8980
αxz	− 1.4680	6.81352
αyz	83.6396	28.4852
α, a.u.	292.7824	17.5023
Δα, a.u.	1080.8721	53.5323
*β*xxx	257.9773	8.1445
*β*xxy	225.4884	33.6499
*β*xyy	579.8797	8.7374
*β*yyy	− 33.4540	− 45.2482
*β*xxz	− 57.6522	− 37.5444
*β*xyz	− 15.9113	31.7805
*β*yyz	− 20.9800	30.0804
*β*xzz	15.7248	7.7288
*β*yzz	− 1.7577	46.5095
*β*zzz	5.2826	− 18.9513
*β*, a.u.	882.5462	63.6244

### Thermal stability

**Fig. 10:**
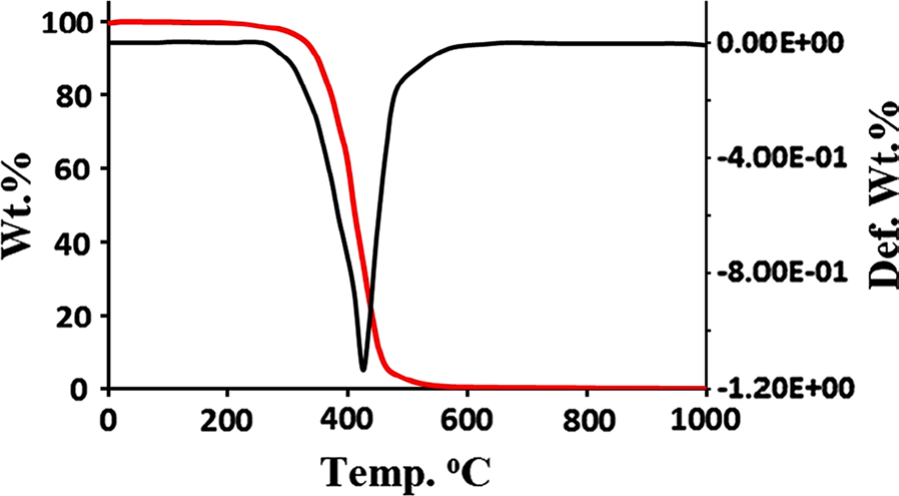
TG/DTG curves

TG/DTG analysis of the desired thiobarbiturate was performed in the temperature range of 0 to 1000 °C in open air atmosphere with heat rate of 5 °C min^−1^, as seen in Fig. 10. Below 380 °C, the compound displayed good thermal stability, and then it thermally decomposed in one step in the range of 380–520 °C, above 520 °C, the compound was completely decomposed with zero mass residue.

## Conclusions

Novel 5-((5-chloro-1*H*-indol-2-yl)methylene)-1,3-diethyl-2-thioxodihydro-pyrimidine-4,6(1*H*,5*H*)-dione via Knoevenagel mild condensation condition. Exo–endo isomerization reaction in the desired molecule was computed, T.S structure and energy level was detected under QTS2 level of calculation. The exo-structure was proven by XRD-analysis measurement, several physical analyses like: CHN-EA, MS, IR, UV–Vis., ^1^H and ^13^C NMR consisted with such seen. The DFT/B3LYP/6-311G(d) structural optimized data were agreed with the XRD-parameters. The exp. XRD-lattice interactions were computed by HSA, MEP map and Mulliken charge, several H-bonds and π–π stacked short interactions were detected. The DFT/6-311G(d) calculations like B3LYP-IR, TD-SCF, HOMO–LUMO, GRD and GIAO NMR reflected a high agreement with their corresponding experimental parameters. NLO-theoretical calculation showed excellent optical properties of the compound, it is even ~ 20 better than urea-reference. The compound TG/DTG analysis revealed a high thermal stability with one step decomposition reaction.

## Experimental

### General

The XRD-data was collected on a Bruker APEX-II D8 diffractometer. The NMR spectra were run in DMSO-*d*_6_ using Jeol-400 spectrometer. All the chemicals were purchased from Sigma.

### Synthesis of 1,3-dimethyl-5-(thien-2-ylmethylene)-pyrimidine-2,4,6-(1*H*,3*H*,5*H*)-trione

A mixture of 1,3-diethyl-2-thioxodihydro-pyrimidine-4,6(1*H*,5*H*)-dione (1.0 mmol) and 5-chloro-1*H*-indole-2-carbaldehyde (1.0 mmol) in 50 mL of distilled water was refluxed and stirred for 5 h until a yellow product was precipitated. Water was decanted, and the yellow product was washed with water then left under an open atmosphere for drying (yield, 85%).

The yellow powder product, with a m.p = 360 °C, was collected; molecular formula C_17_H_16_ClN_3_O_2_S; (Calcd. C, 56.43; H, 4.46 and N, 11.69. Found: C, 56.28; H, 4.41 and N, 11.53). [M^+^] *m/z* = 361.3 (360.2, theoretical). ^1^H NMR (400 MHz, DMSO-*d*_6_): (ppm) 1.2 (m, 6H, 2CH_3_), 4.5 (b, 4H, 2C*H*_*2*_), 7.0–9.0 (4 m, 14H, Ar’s), 9.2 (s, 2H, –HC=N–), 13.1 (s, 1H, –HN–). ^13^C-NMR (100 MHz, DMSO-*d*_6_): (ppm) 13.5 (2C, CH_3_CH_2_), 43.1 (2C, CH_3_CH_2_), 110.8, 116.1, 117.7, 121.8, 123.4, 126.1, 131.5, 128.8, 130.7, 139.8, 145.6, 146.9 (10 signals, 10C, Ar’s), 160.8 (2C, C=O), 173.8 (1C, C=S). FT-IR main vibrations, *V*_N–H_ = 3319 cm^−1^, *V*_C–HAr_ = 3176 cm^−1^, *V*_C–H aliph_ = 2979 cm^−1^, *V*_C=O_ = 1669 cm^−1^, *V*_C=S_ = 1365 cm^−1^, *V*_C=C_ = 1286 cm^−1^.

### Computational details

Hirshfeld surface analysis (HSA) been performed using the CRYSTAL EXPLORER 3.1 program [29]. All Computational calculations of the desired compound were performed by Gaussian 09 software [30]. The molecule optimization geometries, IR vibrations, HOMO/LUMO, TD-SCF, NLO, GRD analysis were carried on DFT/B3LYP level of theory using 6-311G(d, p) base set, NMR chemical shifts were performed at DFT/B3LYP/level of theory and 6-311++G(d,p) base set via adopting GIAO method [31].
